# Tendon-Derived Stem Cell Differentiation in the Degenerative Tendon Microenvironment

**DOI:** 10.1155/2018/2613821

**Published:** 2018-10-28

**Authors:** Chang Liu, Jing-wan Luo, Ke-ke Zhang, Long-xiang Lin, Ting Liang, Zong-ping Luo, Yong-qing Zhuang, Yu-long Sun

**Affiliations:** ^1^Center for Translational Medicine Research and Development, Shenzhen Institute of Advanced Technology, Chinese Academy of Sciences, Shenzhen 518000, China; ^2^Central Laboratory, Dalian Municipal Central Hospital, Dalian 116033, China; ^3^Institute of Orthopaedics, Soochow University, Suzhou 215007, China; ^4^Shenzhen People's Hospital, Shenzhen, Guangdong, China

## Abstract

Tendinopathy is prevalent in athletic and many occupational populations; nevertheless, the pathogenesis of tendinopathy remains unclear. Tendon-derived stem cells (TDSCs) were regarded as the key culprit for the development of tendinopathy. However, it is uncertain how TDSCs differentiate into adipocytes, chondrocytes, or osteocytes in the degenerative microenvironment of tendinopathy. So in this study, the regulating effects of the degenerative tendon microenvironment on differentiation of TDSCs were investigated. TDSCs were isolated from rat Achilles tendons and were grown on normal and degenerative (prepared by stress-deprived culture) decellularized tendon slices (DTSs). Immunofluorescence staining, H&E staining, real-time PCR, and Western blot were used to delineate the morphology, proliferation, and differentiation of TDSCs in the degenerative microenvironment. It was found that TDSCs were much more spread on the degenerative DTSs than those on normal DTSs. The tenocyte-related markers, COL1 and TNMD, were highly expressed on normal DTSs than the degenerative DTSs. The expression of chondrogenic and osteogenic markers, COL2, SOX9, Runx2, and ALP, was higher on the degenerative DTSs compared with TDSCs on normal DTSs. Furthermore, phosphorylated FAK and ERK1/2 were reduced on degenerative DTSs. In conclusion, this study found that the degenerative tendon microenvironment induced TDSCs to differentiate into chondrogenic and osteogenic lineages. It could be attributed to the cell morphology changes and reduced FAK and ERK1/2 activation in the degenerative microenvironment of tendinopathy.

## 1. Introduction

Tendinopathy is common in athletes and people in many occupations involving repetitive work. It is associated with focal tendon tenderness, activity-related pain, and decrease of strength and movement in the affected position. The pathogenesis of tendinopathy remains elusive. There still is a big challenge to prevent tendinopathy and to develop effective treatment for it [1].

Kannus and Jozsa analyzed the histological changes in tendon tissue of 891 tendinopathy patients in 1991 [2]. It was found that over 97 percent of injured tendon tissues had degenerative changes including tendolipomatosis, proteoglycan accumulation, and calcifying tendinopathy, which implied the presence of adipocytes, chondrocytes, or even osteocytes in tendinopathy. However, it was unclear where these nontendinous cells came from. Tendon-derived stem cells (TDSCs) were discovered in tendon tissues in 2007 [3]. Similar to other multipotent stem cells, TDSCs were positive for stem cell-related surface markers such as CD44, CD90, and CD146 and negative for CD34, CD45, and CD106 [3]. They were able to self-renew and had adipogenic, chondrogenic, and osteogenic differentiation potentials. Therefore, the adipocytes, chondrocytes, and osteocytes in tendinopathy were considered to be differentiated from TDSCs [4, 5]. TDSCs could be the culprit for degenerative tendinopathy by undergoing aberrant nontenocyte differentiation into fatty-like, cartilage-like, and bone-like tissues in tendons that compromise tendon structures [6, 7].

Repetitive mechanical loading has generally been considered to be the etiology of tendinopathy [8]. In vivo and in vitro studies performed by Zhang and Wang confirmed that TDSCs differentiated into adipocytes, chondrocytes, and osteocytes when large mechanical stretching was applied [9]. It was found that the microenvironment, where stem cells reside in, regulates the differentiation of stem cells [10, 11]. Nevertheless, nontenocyte differentiation of TDSCs into adipocytes, chondrocytes, and osteocytes in the degenerative microenvironment within tendinopathy has remained unrevealed. Therefore, this study investigated the effects of degenerative tendon microenvironment on the differentiation of TDSCs in vitro to further clarify how TDSCs played their roles in the development of tendinopathy.

Recent discoveries confirmed that complete removal of external stress, also known as stress deprivation, could lead to the degeneration of tendons as well [12–14]. Clinical observations from bedridden patients to astronauts all support this point of view [15]. In addition, when microinjuries in the tendon result from repetitive loading, the unloaded portion of the tendon was stress deprived [16]. And it has been hypothesized that the subsequent stress deprivation conditions resulted from the preconditioned overload are responsible for the development of tendinopathy [16, 17]. Thus, stress deprivation is a relevant model, which simulates some effects of clinical tendinopathy.

In recent years, an extracellular matrix (ECM) from decellularized tendons was isolated, which harbors the biochemical cues of tissue microenvironment including growth factors, proteins, glycosylaminoglycans, and proteoglycans. Besides, they maintain the native ultrastructure and mechanical strength of the tendon tissue to the greatest extent, which provide ideal scaffolds for in vitro study [9, 18, 19].

## 2. Materials and Methods

### 2.1. Isolation of Tendon-Derived Stem Cells (TDSCs)

Approval was obtained from the Institutional Animal Care and Use Committee of Shenzhen Institutes of Advanced Technology, Chinese Academy of Sciences, prior to performing the study. Achilles tendons were harvested from Sprague-Dawley rats (4–6 weeks, male, 160–180 g) (Vital River Laboratory Animal Technology Co., Beijing, China). After the tendon sheaths were removed, the core portions were minced into small pieces. Then the pieces were digested with 4 mg/ml dispase (Sigma-Aldrich, St. Louis, MO, USA) and 3 mg/ml collagenase I (Sigma-Aldrich, St. Louis, MO, USA) in phosphate-buffered saline (PBS) (HyClone, Logan, Utah, USA) solution for 3 hours at 37°C. After adding Dulbecco's Modified Eagle's Medium (DMEM) (HyClone, Logan, Utah, USA) supplemented with 10% FBS (Thermo Fisher Scientific, Pittsburgh, PA, USA), cell suspension was centrifuged at 300*g* for 15 minutes to obtain cell pellets. After cell counting, the cells were inoculated in a 25 cm^2^ culture flask with the density of 500 cells/cm^2^ in DMEM supplement with 10% FBS, 100 U/ml penicillin (HyClone, Logan, Utah, USA), and 100 mg/ml streptomycin (HyClone, Logan, Utah, USA). Approximately 10 days later, TDSC colonies were formed and were isolated by local digestion with 0.25% trypsin (HyClone, Logan, Utah, USA).

### 2.2. Fluorescence-Activated Cell Sorting (FACS) Analysis

To verify the stemness of the cells, TDSCs at passage 2 were incubated with FITC-CD44 (1 : 1000) (BioLegend, San Diego, CA, USA), FITC-CD90 (1 : 8000) (BioLegend), FITC-CD45 (1 : 1000) (BioLegend), and PE-CD106 (1 : 1000) (BioLegend) in the dark at 4°C for 20 minutes. After being washed three times with PBS, TDSCs were analyzed by a BD FACSCanto II flow cytometer (BD Biosciences, San Jose, CA, USA).

### 2.3. Multidifferentiation Potential of TDSCs

TDSCs were inoculated in a six-well plate with 500 cells/cm^2^ density, and adipogenic, chondrogenic, and osteogenic induction media (Thermo Scientific, Pittsburgh, PA, USA) were added. The media were changed every other day. Two weeks later, TDSCs were fixed with 4% paraformaldehyde (Sigma-Aldrich, St. Louis, MO, USA) at room temperature for 15 minutes. After being washed with deionized water, cells were stained with Oil Red O (Solarbio Life Science, Beijing, China), Safranin O (Solarbio life science), and Alizarin Red S (Solarbio Life Science). The stained cells were verified by inverted light microscope (CKX41, Olympus, Tokyo, Japan).

### 2.4. Stress-Deprived Tendon Model Construction

Rat Achilles tendons were harvested and maintained under stress-deprived conditions, based on a previously reported study with some modifications [13]. In brief, the fresh Achilles tendons were cultured in DMEM supplemented with 10% FBS, 100 U/ml penicillin, and 100 mg/ml streptomycin and incubated at 37°C in a humidified atmosphere of 5% CO_2_ in air for up to 4 weeks. The medium was changed every other day. The stress-deprived tendons were collected as degenerative tendons hereafter in this study.

### 2.5. Live/Dead Staining

Normal and degenerative tendons were stained with live/dead staining working solution (Life Technologies, Pittsburgh, PA, USA) composed of 2 *μ*M calcein AM and 4 *μ*M ethidium homodimer-1 at 37°C for 0.5 h. After being washed with PBS, the cells within the depth of 70 *μ*m of the tissue surface were observed using confocal laser scanning microscopy (LSM 880, Zeiss, Oberkochen, Germany).

### 2.6. H&E Staining

Normal and degenerative tendons were fixed with 4% paraformaldehyde, dehydrated with 30% sucrose and embedded with OCT (Sakura Finetek, Torrance, CA, USA), and frozen at −80°C before section. Cryosections were cut at 5 *μ*m thickness and stained with hematoxylin and eosin (Sakura Finetek, Torrance, CA, USA) to observe cell morphology and cell number. Histological samples were imaged using an upright microscope (BX53, Olympus, Tokyo, Japan).

### 2.7. Preparation of Decellularized Tendon Slices (DTSs)

DTSs were prepared according to the published protocols with some modifications [18]. In brief, normal and degenerative tendon tissues were embedded with OCT, frozen at −80°C, fixed to the cutting base plate of a cryostat (CM1950, Leica, Nussloch, Germany), and longitudinally cut into slices with a thickness of 100 *μ*m. After removing the OCT by washing with PBS 3 times for 30 minutes, tendon slices were frozen in liquid nitrogen for 2 minutes and thawed in normal saline for 10 minutes at 37°C for 5 cycles. Then the tendon slices were incubated in 200 *μ*g/ml RNase and 400 *μ*g/ml DNase (Sigma-Aldrich, St. Louis, MO, USA) for 4 hours at 37°C, followed by washing with PBS at room temperature with gentle agitation. Finally, each side of the DTSs was sterilized with UV radiation for 2 hours for further usage.

### 2.8. DNA Staining and Extraction

Normal and degenerative DTSs were incubated with DAPI at room temperature for 15 minutes, followed by washing with PBS 3 times. The cell nucleus was observed by an inverted fluorescence microscope (Eclipse, Nikon, Tokyo, Japan). Total DNA of normal and degenerative DTSs was extracted by TIANamp Genomic DNA Kit (TIANGEN Biotech Co., Beijing, China) following the manufacturer's instructions. DNA content was measured with an ultraviolet spectrophotometer (NanoDrop 2000, Thermo Fisher Scientific, Pittsburgh, PA, USA).

### 2.9. Elastic Modulus of Normal and Degenerative DTSs

The elastic modulus of normal and degenerative DTSs was measured by atomic force microscope (AFM) (Dimension ICON, Bruker, Billerica, Massachusetts, USA). Oxide-sharpened, square-based pyramidal silicon-nitride tips with a borosilicate glass sphere at a radius of 5 *μ*m glued onto the V-shaped silicon-nitride cantilevers with a nominal spring constant of 0.06 N/m were employed. Cyclic load-displacement curves were recorded at 6 different sites on the sample surface. In all cases, the DTSs were immersed into pH 7.4 PBS solution.

### 2.10. Cell Viability Analysis

Cell viability was detected using a Cell Counting Kit-8 (CCK8) assay (Dojindo Laboratories, Kumamoto, Japan), according to the manufacturer's instructions. Briefly, TDSCs were inoculated on normal and degenerative DTSs with a density of 1000 cells/cm^2^. At selected time points, TDSCs were incubated with DMEM plus 10% (*v*/*v*) CCK8 at 37°C for 2 h, then 200 *μ*l supernatant was used to measure the absorbance. The absorbance at 450 nm with a reference wavelength of 630 nm was recorded using a microplate reader (FC, Thermo Fisher Scientific, Pittsburgh, PA, USA).

### 2.11. F-Actin Staining

TDSCs on normal and degenerative DTSs were fixed with 4% paraformaldehyde at room temperature for 15 minutes, followed by washing with PBS 3 times, then treated with PBS containing 0.1% Tween-20 (Sigma) and 1% goat serum (Thermo) for 15 minutes, and washed with PBS 3 times. Finally, the TDSs were incubated with Flash Phalloidin Green 488 (1 : 20) (BioLegend) and DAPI (Sigma) at 4°C in the dark for 30 minutes. Photos were taken by an inverted fluorescence microscope (Eclipse, Nikon).

### 2.12. Quantitative Real-Time Reverse Transcription-Polymerase Chain Reaction

Total RNA was isolated using an RNA purification kit (Corning, New York, USA) according to the manufacturer's instructions. Reverse transcription (RT) was performed using a PrimeScript RT Reagent Kit (TaKaRa, Shiga, Japan). Real-time polymerase chain reaction (PCR) was carried out with SYBR Premix Ex Taq (Perfect Real Time) (Takara). PCR amplification and fluorescence detection were performed using a LightCycler® 96 System (Roche, Basel, Switzerland). The primers used in this study were listed in Table S. GAPDH was used as an internal control. The results were presented as the calculated comparative expression ratios of the target sample to control group for each sample using the C_T_ method (2^−ΔΔC_T_^).

### 2.13. Western Blot

TDSCs were lysed in lysis buffer containing protease and phosphatase inhibitors (Keygentec, Nanjing, China). Protein concentration was quantified by BCA kit (Keygentec), and an equal amount of protein was loaded in each lane. Constant voltage electrophoresis was carried out with 10% polyacrylamide gels. Then the proteins were transferred to polyvinylidene fluoride (PVDF) membranes (Merck, Massachusetts, USA). PVDF membranes were blocked with 3% BSA (Sigma) and hybridized with ERK1/2 (Santa Cruz Biotechnology, Dallas, TX, USA), phospho-ERK1/2 (Thr202/Tyr204) (Santa Cruz Biotechnology), FAK (Santa Cruz Biotechnology), phospho-FAK (Tyr397) (Santa Cruz Biotechnology), and *β*-actin (Santa Cruz Biotechnology) primary antibodies overnight at 4°C. After being washed with TBST (Keygentec), the PVDF membranes were incubated with secondary antibodies (ZSGB-bio, Beijing, China) at room temperature for 30 minutes, followed by washing with TBST. Finally, the PVDF membranes were covered with 3,3-diaminobenzidine (DAB) for the display of specific protein bands.

### 2.14. Statistical Analysis

All individual experiments were performed at least three times, with three replicates. Data were expressed as means ± standard deviation (SD). The significance of differences between two groups were determined using unpaired Student's *t*-tests. Differences among more than two experimental groups were evaluated by one-way ANOVA. Differences were considered significant at *P* < 0.05.

## 3. Results

### 3.1. Characterization of TDSCs

**Figure 1:**
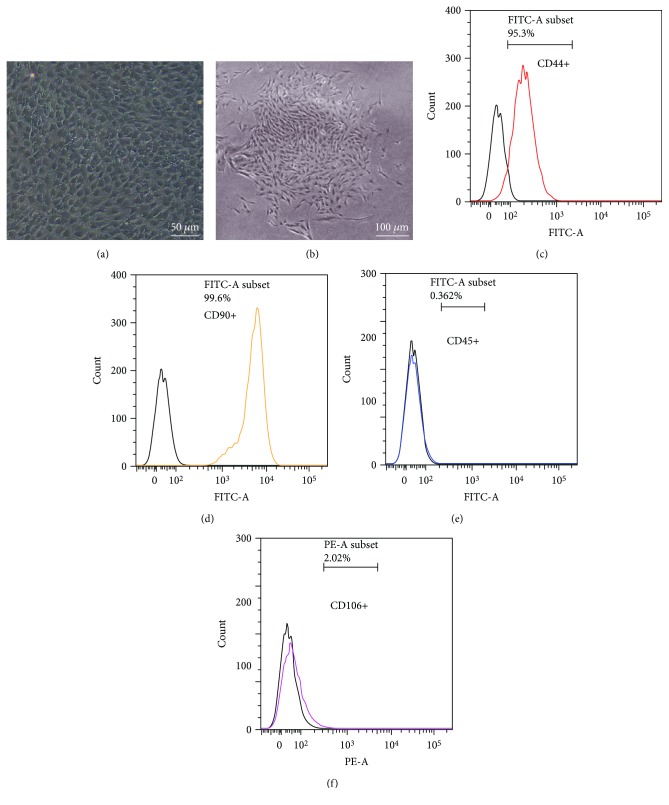
The morphology and stemness characteristics of TDSCs: (a) morphology and (b) colon-formation ability of TDSCs and flow cytometric analysis of (c) CD44, (d) CD90, (e) CD45, and (f) CD106 expression of TDSCs.

**Figure 2:**
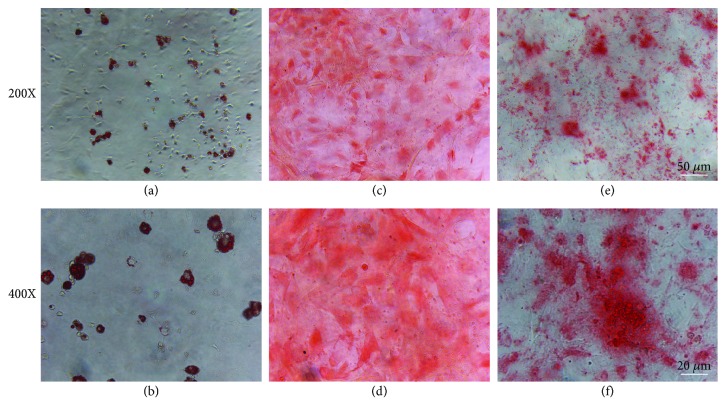
Multidifferentiation potential of TDSCs: (a-b) Oil Red O staining, (c-d) Safranin O staining, and (e-f) Alizarin Red S staining.

TDSCs, from rat Achilles tendon, showed cobblestone-shaped morphology (Figure 1(a)) and had clone-forming ability (Figure 1(b)). In addition, the flow cytometric analysis showed that 95.3% of cells were positive for CD44 (Figure 1(c)) and 99.6% of cells were positive for CD90 (Figure 1(d)), which are stem cell-related markers. In addition, TDSCs were negative for leukemia cell marker CD45 and endothelial cell marker CD106 (Figures 1(e) and 1(f)). Furthermore, the multilineage differentiation assay confirmed that TDSCs were able to differentiate into adipocytes, chondrocytes, and osteocytes, which were demonstrated by Oil Red O (Figures 2(a) and 2(b)), Safranin O (Figures 2(c) and 2(d)), and Alizarin Red S (Figures 2(e) and 2(f)) staining, respectively, after induction.

### 3.2. Degenerative Tendon Construction

**Figure 3:**
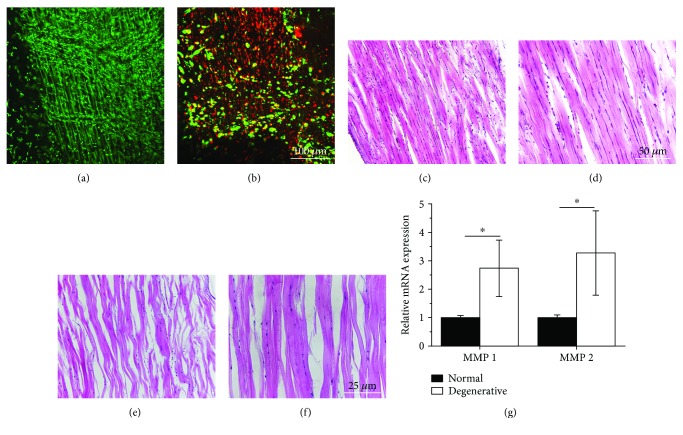
Live/dead, H&E staining, and MMP expressions of normal and degenerative tendons. Live/dead staining of (a) normal and (b) degenerative tissues. H&E staining of (c-d) normal and (e-f) degenerative tendons. (g) Comparison of expressions of MMP 1 and MMP 2 in normal and degenerative tendons (*n* = 8). ^∗^ *P* < 0.05, compared with normal tendons.

The degenerative tendon was produced by stress-deprived culture of rat Achilles tendons ex vivo for 4 weeks. Live/dead staining showed that almost all cells have good viability within a fresh tendon (Figure 3(a)); however, a large number of cells in the stress-deprived tendon died (Figure 3(b)). H&E staining confirmed that tendon cells had elongated morphology in the normal tendon (Figures 3(c) and 3(d)). While in the degenerative tendon, more rounded cells, a loose collagen structure, and increased gaps between the collagen fibers were observed (Figures 3(e) and 3(f)). In addition, the number of cells decreased significantly in the degenerative tendon compared with the normal tendon (Figures 3(e) and 3(f)). Higher expressions of matrix metalloproteinase (MMP), such as MMP 1 and MMP 2, were found in the tendinopathic tendons. MMP 1 is one of the collagenases with proteolytic activity in type I collagen, the most abundant collagen in tendons. This study found that TDSCs on the degenerative tendon tissues expressed significantly higher levels of MMP 1 and MMP 2 compared to those on normal tendons. It suggested the degradation of type I collagen in the degenerative tendon (Figure 3(g)). The above results indicated the degeneration occurred in the Achilles tendon under stress-deprived culture.

### 3.3. Preparation of Decellularized Tendon Slices (DTSs)

**Figure 4:**
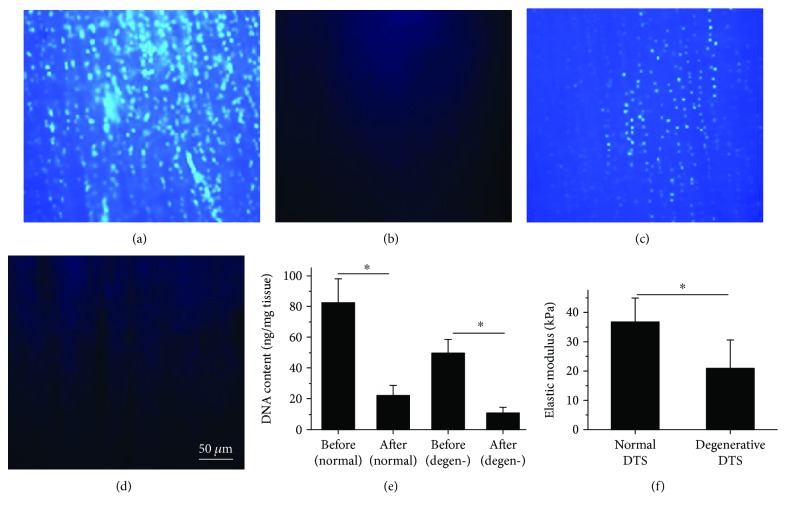
Decellularization of normal and degenerative tendons. DAPI staining of (a-b) normal and (c-d) degenerative DTSs before and after decellularization, respectively. (e) DNA content of normal and degenerative DTSs before and after decellularization. ^∗^ *P* < 0.05, compared with the after decellularization group. The error bars represent the standard deviation of measurements for 5 separated samples (*n* = 5). (f) Elastic modulus of normal and degenerative DTSs. ^∗^ *P* < 0.05, compared with normal DTSs. The error bars represent the standard deviation of measurements for 6 points in 3 separated samples (*n* = 18).

A repetitive freeze/thaw procedure with nuclease treatment on normal and degenerative tendon tissues was applied to remove the resident cells and to produce extracellular matrix scaffolds of normal and degenerative tendons. After decellularization, the number of cells in normal (Figures 4(a) and 4(b)) and degenerative (Figures 4(c) and 4(d)) tendons deceased significantly. Furthermore, DNA content in normal and degenerative tendons were significantly lower after decellularization (Figure 4(e)).

### 3.4. Matrix Stiffness of Normal and Degenerative DTSs

The compressive elastic modulus can be used to represent the matrix stiffness of tendon tissue. The matrix stiffness of degenerative DTS was 20.98 ± 9.57 kPa, significantly lower than that of normal DTS (36.79 ± 7.39 kPa) (Figure 4(f)), which indicated that the degeneration was associated with the lower stiffness of tendon microenvironment.

### 3.5. Cell Viability and Proliferation of TDSCs

**Figure 5:**
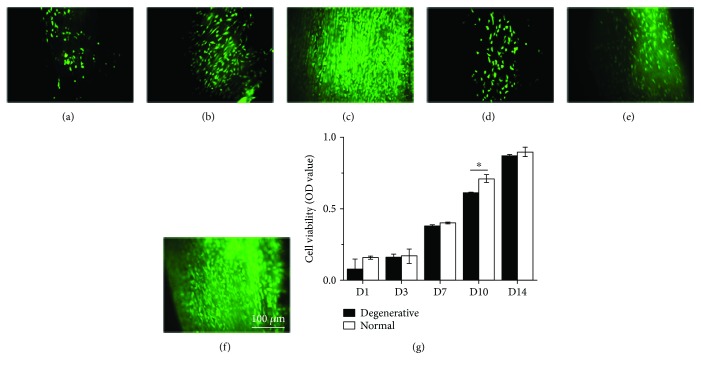
Live/dead staining and proliferative ability of TDSCs on normal and degenerative DTSs. Live/dead staining of TDSCs on normal DTSs at (a) day 1, (b) day 7, and (c) day 14. And live/dead staining of TDSCs on degenerative DTSs at (d) day 1, (e) day 7, and (f) day 14. (g) Proliferative ability assessment of TDSCs on normal and degenerative DTSs. ^∗^ *P* < 0.05, compared with normal DTSs. The error bars represent the standard deviation of measurements for 3 replicates of 3 separated samples (*n* = 9).

During the culture period of 14 days, TDSCs were well attached to the surfaces and maintained good viability on normal (Figures 5(a)–5(c)) and degenerative (Figures 5(d)–5(f)) DTSs. From the 7th day, TDSCs started to show elongated spindle morphology and were aligned along the direction of collagen fibers on normal DTSs (Figures 5(b) and 5(c)). However, the shape of TDSCs on degenerative DTSs was more spread out (Figures 5(e) and 5(f)). Similar to the microscopic observation, the CCK8 assay revealed that during the culture period of 14 days, the viability of TDSCs on normal and degenerative DTSs was significantly increased (Figure 5(g)). The proliferation of TDSCs had the same trend on normal and degenerative DTSs. However, TDSCs on normal DTSs showed higher viability since day 7, and the difference was significant on day 10 (Figure 5(g)).

### 3.6. F-Actin Fiber Formation and Distribution

**Figure 6:**
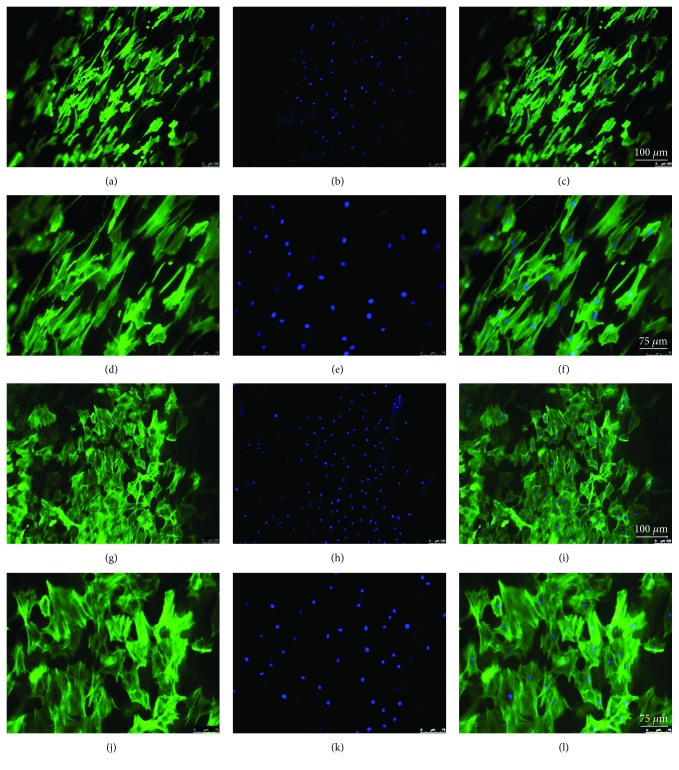
F-actin (labeled with Alexa488-phalloidin) and nucleus (labeled with DAPI) staining and merged images of TDSCs. TDSCs on (a–f) normal and (g–l) degenerative DTSs magnified 100 and 200 times, respectively.

F-actin staining was performed to investigate the alteration of TDSC cytoskeleton organization between normal and degenerative DTSs. TDSCs were aligned, with disorganized actin filaments on normal DTSs (Figures 6(a)–6(f)). However, TDSCs adopted polygonal shapes with bundled actin fibers on degenerative DTSs (Figures 6(g)–6(l)).

### 3.7. TDSC Differentiation on DTSs

**Figure 7:**
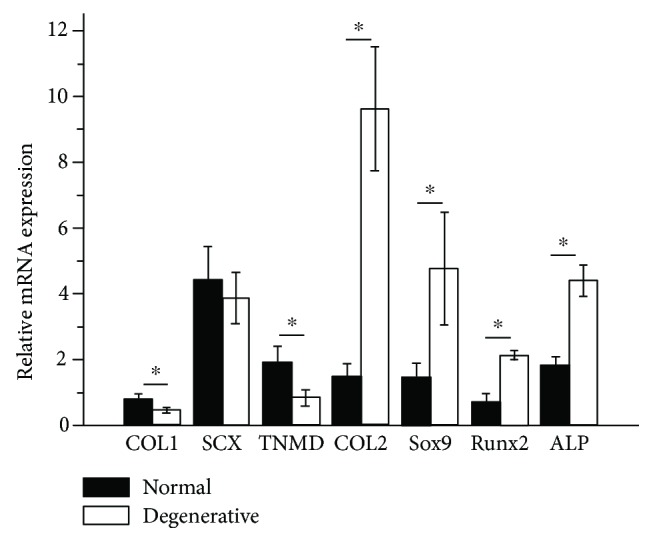
The differentiation of TDSCs toward tenogenic, chondrogenic, and osteogenic lineages. Relative gene expression of tenocyte markers COL1, SCX, and TNMD, chondrocyte markers COL2 and SOX9, and osteocyte markers Runx2 and ALP. The results were represented as the calculated comparative expression ratios of normal and degenerative DTS groups to the culture dish group. ^∗^ *P* < 0.05, compared with normal DTSs. The error bars represent the standard deviation of measurements for 2 replicates of 8 separated samples (*n* = 16).

In order to understand the effects of degenerative tendon microenvironment on the differentiation of TDSCs, the expressions of tenogenic, chondrogenic, and osteogenic markers were detected. Tenocyte markers, COL1, SCX, and TNMD, were highly expressed on normal DTSs than on degenerative DTSs, especially COL1 and TNMD (Figure 7). On the contrary, chondrogenic markers COL2 and SOX9 and osteogenic markers Runx2 and ALP were expressed at higher levels on degenerative DTSs than normal DTSs (Figure 7). The results indicated that a normal tendon matrix tended to induce the differentiation of TDSCs into tenocytes. However, TDSCs in the degenerative microenvironment had a tendency to differentiate into chondrocytes and osteocytes.

### 3.8. Expression of Integrin *β*1, FAK, and ERK

**Figure 8:**
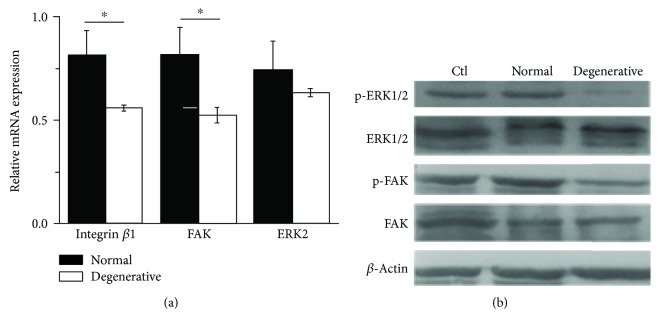
Gene and protein expression of FAK and ERK: (a) Relative gene expressions of integrin *β*1, FAK, and ERK and (b) Western blot from whole-cell lysates showing expressions of phosphorylated FAK^Tyr397^ and phosphorylated ERK1/2^Thr202/Tyr204^ in TDSCs on a culture dish (as the control) and normal and degenerative DTSs. ^∗^ *P* < 0.05, compared with normal DTSs. The error bars represent the standard deviation of measurements for 2 replicates of 8 separated samples (*n* = 16).

The expression of FAK and ERK1/2 was evaluated both at gene and protein levels. It was found that the expression of integrin *β*1, FAK, and ERK at the mRNA level was lower on degenerative DTSs than on normal ones, and the differences of integrin *β*1 and FAK were significant (Figure 8(a)). In addition, phosphorylated FAK on Tyr397 and phosphorylated ERK1/2 on Thr202 and Tyr204 were significantly decreased on degenerative DTSs than on normal DTSs (Figure 8(b)).

## 4. Discussion

It was hypothesized that the erroneous differentiation of TDSCs to chondrocytes or osteoblasts leads to chondrometaplasia and ossification in tendinopathy [20]. However, it is unclear what factors induce TDSCs to differentiate into the tenogenic, adipogenic, chondrogenic, and osteogenic phenotypes. In this study, an in vitro degenerative tendon model, which was constructed by stress deprivation, was used to investigate the tenogenic and nontenogenic differentiation of TDSCs in the degenerative microenvironment.

Mechanical loading is essential for maintaining the homeostasis of tendons. However, when the microinjuries occurred in tendons, the partially unloaded area, which was stress deprived, was created at the same time. Stress deprivation has been confirmed to lead to the degeneration of tendons, including changes in collagen organization and mechanical properties similar to the effects of tendinopathy [21, 22]. So, at least to some extent, the degenerative tendon model produced by stress-deprived culture could mimic the degenerative microenvironment of tendinopathy.

Shi et al. [23] found that elongated TDSCs exhibited enhanced gene expression of tenogenic markers compared with the cells which were spread out. However, the elongated cell shape inhibited chondrogenic and osteogenic differentiation of TDSCs. Our results were in accordance with the previous study; on normal DTSs, TDSCs adopted elongated shape and expressed a significantly higher level of tenogenic markers. On the contrary, on degenerative DTSs, TDSCs were more spread and significantly enhanced the expression of chondrogenic and osteogenic markers. These results indicated that in the degenerative microenvironment, TDSCs tended to be spread and differentiated toward chondrogenic and osteogenic phenotypes other than the tenogenic phenotype, which might lead to calcification or even ossification identified in tendinopathy.

Matrix stiffness, a biophysical cue in the cell microenvironment, played important roles in stem cell differentiation [24–26]. Mesenchymal stem cells (MSCs) [27], adipose-derived stem cells (ADSCs) [28], and umbilical cord mesenchymal stem cells (UCMSCs) [11], which were cultured on substrates with different stiffness, have been shown to possess diverse lineage commitments. MSCs would differentiate toward bone, muscle, or neuronal lineages as they grew on substrates with high, medium, or low stiffness, respectively [29]. The mechanical strength of the injured tendon tissues decreased significantly [30, 31]. The tissue stiffness would decline from 185 kPa to 60.3 kPa [31]. In this study, we found that the matrix stiffness of degenerative DTSs was significantly lower than the normal ones. So the matrix stiffness could be involved in the regulation of TDSC differentiation.

Matrix stiffness was sensed by integrin *β*1 on the cell membrane, which mediated the interaction between cell and ECM [32]. It was shown that TDSCs on normal DTSs had a higher expression level of integrin *β*1 compared with that on degenerative DTSs. Similar findings were shown by Gershlak and Black, in which the MSCs growing on the collagen I-coated PA gels displayed a general increase in the average number of the *β*1 integrin subunit with increasing stiffness [32].

Focal adhesion kinase (FAK) plays an important role in mediating mechanotransduction pathways. It mediates the interactions between cell-cell and cell-matrix. Furthermore, FAK affects intracellular signals through the extracellular signals. Extracellular signal-regulated kinase 1/2 (ERK1/2) is a well-known downstream signaling molecular of FAK and a force-activated protein kinase [33]. The FAK-ERK1/2 signaling pathway was confirmed to be involved in many cell activities including cell proliferation and cell differentiation [34]. Shih et al. [35] found that when FAK or ERK1/2 activation was inhibited, the differentiation of MSCs toward the osteogenic lineage was blocked. However, Wang et al. obtained an opposite result: the phosphorylated activation of ERK1/2 leads to the inhibition of osteogenic differentiation of MSCs [36]. In this study, similar results with Wang's were found: TDSCs on normal DTSs had significantly enhanced phosphorylated ERK1/2 expression, while osteogenic lineage-related markers Runx2 and ALP were lower expressed. However, TDSCs on degenerative DTSs expressed reduced phosphorylated ERK1/2, but osteogenic markers were highly expressed.

The morphology of cells was determined by the cytoskeleton, a structure subject to extracellular stress. The cytoskeleton connects with the ECM through focal adhesions (FA). Therefore, the actin cytoskeleton is continuously stabilized and destabilized by FAK-regulated processes. Several studies showed that when ADSCs or MSCs grew on substrates of different stiffness, the spreading area increased and more F-actin stress fibers formed on the substrate with higher stiffness [26, 28]. Nevertheless, in this study, it was found that the degenerative DTSs with lower stiffness enhanced the formation of F-actin stress fibers. There was evidence that F-actin fiber formation was promoted when FAK-ERK1/2 signaling was inhibited in liver cancer stem cells [33]. Our results were in accordance with theirs that the phosphorylation of FAK and ERK1/2 were reduced on degenerative DTSs, so that F-actin formation was increased compared with the normal DTSs with higher stiffness.

Mechanical loading constantly acts on tendons under physiological conditions. The differentiation of TDSCs toward tenocytes and nontenocytes was reported to be affected by the mechanical loading. When loaded with a 4% stretch, TDSCs highly expressed tenocyte-related markers COL1 and TNMD, while nontenocyte markers LPL, Sox9, Runx2, and Osterix were not affected [9]. However, as TDSCs were exposed to 8% stretching, both tenocyte- and nontenocyte-related genes were upregulated [9]. This study only focused on the different behaviors of TDSCs on degenerative DTSs without mechanical loading. The mechanical loading will be included in the future study.

Finally, besides the biophysical cues, the degenerative tendon experienced a series of biochemical changes, including the growth factor or ECM protein loss during the stress-deprived culture period. Tuan et al. found that the soluble fraction of tendon ECM could enhance the differentiation of ADSCs into tenogenic lineage [37], which indicated that the soluble ECM was also important for tenogenic differentiation. We cannot exclude that the observed differences were influenced by the ECM protein loss in the degenerative tendon microenvironment in this study. We will investigate the effect of biochemical changes in the degenerative tendon on the differentiation of TDSCs in the following study. Nevertheless, the results we found in this study would provide evidence that the degenerative microenvironment could induce TDSCs to differentiate into chondrogenic and osteogenic lineages. The regulating effect might be attributed to the cell morphology change and the reduced FAK and ERK1/2 activation in the degenerative microenvironment with reduced matrix stiffness.

## 5. Conclusions

It is important to understand the role of TDSCs in the pathogenesis of tendinopathy and the mechanisms contributing to their erroneous differentiation for the management of tendinopathy. This study demonstrated that TDSCs undergo erroneous differentiation toward chondrogenic and osteogenic lineages on degenerative DTSs. The underlying mechanism might be attributed to the degenerative microenvironment with the decrease of matrix stiffness, which resulted in the change of cell morphology and the reduced activation of FAK and ERK1/2.

## Data Availability

The data used to support the findings of this study have been deposited in the figshare repository (10.6084/m9.figshare.6210050). The primer sequence data used to support the findings of this study are included within the supplementary information file.

## Supplementary Materials

Supplementary Materials Table S: real-time PCR primers. The table shows the primers used for real-time PCR analysis in this study; GAPDH was used as an internal control.
