# Supplementary material for: Tendon-Derived Stem Cell Differentiation in the Degenerative Tendon Microenvironment
Source: Stem Cells Int. 2018 Oct 28;2018:2613821. doi: 10.1155/2018/2613821 (PMC6230403; doi:10.1155/2018/2613821)
Supplement: Supplementary Materials — Table S: real-time PCR primers. The table shows the primers used for real-time PCR analysis in this study; GAPDH was used as an internal control. [file 2613821.f1.docx]

Table S Real time PCR primers. The table shows the primers used for Real time PCR analysis in this study, GAPDH was used as an internal control.

|  | Sense (5’-3’) | Anti-sense (5’-3’) |
| --- | --- | --- |
| GAPDH | AAGAAACCCTGGACCACCCAGC | TGGTATTCGAGAGAAGGGAGGG |
| COL1 | CATCGTGGCTTCTCTGGTC | ACCGTTGAGTCCATCTTTGC |
| SCX | AGAACACCCAGCCCAAACA | CGGTCTTTGCTCAACTTTCT |
| TNMD | GTCACATTCTAAATGCAGAAG | CTCCCCCAAAACAGGACAAT |
| COL2 | GACCTGCCGGTGAACAAG | GGTACCAGGTTCTCCATCTCT |
| SOX9 | CTCTCCTAACGCCATCTTCAAG | ACGTCTGTTTTGGGAGTGG |
| RUNX2 | TCTTCCCAAAGCCAGAGCG | TGCCATTCGAGGTGGTCG |
| ALP | CCCGAGTGCTTTGTGTGTGCTG | CCGCCGGTGTTCGTGTGTG |
| Integrin β1 | AAAATGGACGAAAGTGCTCTAAC | TGGGACTTGCTGGGATGC |
| FAK | CCAAGTTCGAGTACTAAGACTCACC | AAATCCATAGCAGGCCACGTGC |
| ERK | AGAGATCATGCTTAACTCCAAG | TTCATGTTAATGATACAATTTAGGTCCTC |
